# Implementing physics-informed neural networks with deep learning for differential equations

**DOI:** 10.3389/frai.2026.1717117

**Published:** 2026-02-23

**Authors:** Frank Emmert-Streib, Shailesh Tripathi, Amer Farea, Andreas Holzinger

**Affiliations:** 1 Predictive Society and Data Analytics Lab, Faculty of Information Technology and Communication Sciences, Tampere University, Tampere, Finland; 2 College of Health and Life Sciences, Hamad Bin Khalifa University, Doha, Qatar; 3 Josef Ressel Centre for Data-Driven Business Model Innovation, University of Applied Sciences Upper Austria, Steyr, Austria; 4 Human-Centered AI Lab, Institute of Forest Engineering, Department of Ecosystem Management, Climate and Biodiversity, BOKU University, Vienna, Austria

**Keywords:** data-driven scientific machine learning, forward problem, inverse problem, ordinary differential equation, physics-aware machine learning, physics-informed neural network

## Abstract

Physics-aware machine learning integrates domain-specific physical knowledge into machine learning models, leading to the development of physics-informed neural networks (PINNs). PINNs embed physical laws directly into the learning process, enabling interpretable and physically consistent solutions to complex problems. However, the practical use of PINNs presents challenges and their applications are complex. Therefore, in this paper, we demonstrate the implementation of PINNs for systems of ordinary differential equations (ODEs), an area that is often overlooked by the physics community, which typically focuses on partial differential equations. We discuss two key challenges: the inverse problem, which involves estimating unknown parameters of ODEs, and the forward problem, which provides an approximate solution to ODEs. To provide practical insights into PINNs, we present two case studies based on a Python implementation using DeepXDE. Drawing on these studies, we discuss key challenges and identify promising directions for future research in PINN-based implementation frameworks.

## 1 Motivation and significance

Physics-aware machine learning, physics-inspired machine learning, and physics-informed machine learning refer to the integration of physical principles within machine learning. *Physics-Aware Machine Learning* integrates domain-specific physical knowledge directly into machine learning models, aiming to make the model “aware” of underlying physical principles or constraints. Physics-aware models typically incorporate physical laws or principles to improve interpretability and robustness, especially in contexts where data alone may be insufficient (Cheng et al., 2023). *Physics-inspired machine learning* leverages concepts, structures, and/or processes from physics to design or structure machine learning models, without necessarily embedding specific physical laws of the target system. “Inspiration” could come from physical concepts such as energy minimization, symmetry, or physical architectures that mimic neural networks (such as models inspired by quantum Zhu et al., 2023). *Physics-Informed Machine Learning (PIML)*, finally, involves embedding known physical equations, like differential equations, directly into machine learning algorithms. This approach ensures that the learned model adheres to specific physical laws, often by integrating these laws as constraints within the model's loss function or structure.

A key approach within PIML is to use a physics-informed neural network (PINN), where physical laws are embedded into neural networks, constraining the learning process to physically plausible solutions to guide the learning process, allowing the model to predict solutions that are consistent with known physics.

A PINN encodes model equations, like partial differential equations (PDEs), as part of the neural network, and can solve PDEs, fractional equations, integral-differential equations, and stochastic PDEs. This multitask learning approach requires the neural network to fit the observed data and reduce a residual PDE (Cuomo et al., 2022). Consequently, PINNs are both effective and efficient for ill-posed and inverse problems, and combined with domain decomposition, they are scalable to large problems (Karniadakis et al., 2021; Farea et al., 2024).

By encoding these constraints directly into the loss function, PINNs reduce the dependency on labeled data and are effective for applications in fields such as fluid dynamics, climate science, and structural engineering, where acquiring comprehensive datasets may be costly or infeasible. Moreover, physics-aware machine learning can address multiscale modeling challenges, mitigate training costs through optimized loss functions, and enable the handling of unstructured data like adaptive meshes. As PIML models also address uncertainty quantification and propagation, they offer more reliable predictions, essential for critical applications in physics, engineering, and the natural sciences (Wu et al., 2023; Cai et al., 2021; Lawal et al., 2022).

Interpretability is a crucial aspect of physics-aware machine learning, as it allows researchers and practitioners to understand how model predictions align with known physical principles, thereby enhancing trust and usability in scientific and engineering applications (Hudec et al., 2021). In general, machine learning models—especially deep neural networks—often function as “black boxes,” which limits their reliability in critical fields, where understanding the reasoning behind predictions is essential for model validation and decision-making. Physics-aware machine learning addresses this by embedding physical knowledge directly into the model, facilitating interpretability and enabling researchers to examine how well the model respects known laws of nature, such as conservation laws or symmetry constraints. Currently, as interpretability becomes increasingly important for addressing various complex problems, PINNs offer several advantages: they can solve complex differential equations and approximate highly nonlinear functions; they do not require large datasets, relying instead on boundary or initial conditions; and they are effective in solving inverse problems and parameter estimation in complex systems (Cho et al., 2023).

In this paper, we address several issues that have received limited attention in the current state of the art. Our contribution is primarily methodological and conceptual, focusing on implementation and critical discussion. While physics-informed neural networks (PINNs) have been developed largely within physics, with an emphasis on partial differential equations (PDEs), systems of ordinary differential equations (ODEs) introduce distinct challenges that are not adequately covered by existing approaches. Moreover, although PDEs are central to many physical applications, ODEs play a dominant role in fields such as epidemiology (Brauer et al., 2019), biology and systems biology (Ingalls, 2013), and economics (Tsoularis, 2021).

Consequently, our article highlights these underexplored topics by also focusing on applications outside of physics, which further emphasizes the importance and versatility of physics-aware machine learning.

## 2 Background and related work

PINNs differ fundamentally from traditional neural networks, which often act as surrogate models based purely on empirical data across diverse input-output scenarios (Sallam et al., 2021; Radman et al., 2022; Smolander et al., 2019; Lucor et al., 2021).

Instead of relying solely on data correlations, PINNs integrate physical laws—typically represented as differential equations—directly into the neural network framework. This approach enables PINNs to serve as powerful tools for addressing two key classes of problems: the *forward problem*, which involves predicting system responses based on known parameters, and the *inverse problem*, where the goal is to infer unknown model parameters from observed data. By combining data-driven learning with physical constraints, PINNs not only improve predictive accuracy but also enhance interpretability, making them especially valuable in fields where adhering to physical laws is critical.

Interestingly, despite the popularity of PINNs, their adoption in fields outside of physics has been somewhat limited. This could be due to several reasons. First, there may be an impression that PINNs need to be used with partial differential equations (PDEs) (Meng et al., 2023; Peng et al., 2022; Sun and Feng, 2023; Fu et al., 2023), whilst many applications outside physics are based on ordinary differential equations (ODEs) or systems thereof. However, PINNs are not limited to PDEs and can also be used for systems of ODEs. Second, technical challenges related to the usability of the model may also hinder wider adoption because several steps need to be interconnected with each other in a problem-specific manner. This means that the implementation is more complex than a standard neural network and requires tools and methods that provide greater flexibility, user-friendly modules, and functions that can be effectively applied to the problems. Additionally, several diagnostic capabilities within a minimalistic code framework are needed. The existing literature discusses, in general, various challenges associated with the implementation of Physics-Informed Neural Networks (PINNs). These challenges include training PINNs with fast convergence and high accuracy, which is complicated by multiple loss functions and the presence of high-dimensional non-convex functions (Liu et al., 2023).

Overfitting remains a significant concern (Doumèche et al., 2023), along with the need to maintain architectural simplicity to avoid overparameterization and to balance the number of sampling points against the complexity of the problem for convergence to the global minimum (Liu et al., 2023; Wang et al., 2022), and to address multiscale problems (Wang et al., 2024). Moreover, there is a need to develop tools that facilitate the solving of differential equations using low-code solutions (Matthews and Bihlo, 2024). Optimizing collocation and experimental points is also important for achieving accurate solutions in PINNs while minimizing computational costs; these points are essential for enforcing the differential equations and initial or boundary condition, with experimental points serving as queries for ground truth values (Lau et al., 2024). Incorporating flexibility into the implementation, such as by adding physical loss weights and adaptive hyperparameters to the activation function, enhances the effectiveness of PINNs (Batuwatta-Gamage et al., 2023).

Two recent examples round off our state-of-the-art overview: Ren et al. (2024) introduced a physics-informed neural network (PINN) for seismic wave modeling in semi-infinite domains, addressing computational challenges associated with large geophysical domains. The proposed PINN incorporates an absorbing boundary condition as a soft regularizer, enabling accurate modeling without labeled data. To enhance scalability and accuracy, the model uses a temporal domain decomposition strategy and an innovative surrogate modeling approach for parametric seismic loading. Numerical experiments demonstrate the model's high solution accuracy across various material distributions, highlighting its potential for versatile seismic applications. Kasiraju et al. (2025) introduces an open-source Python-based Parameter Estimation Tool, petBOA, which utilizes Bayesian Optimization (BO) for gradient-free parameter estimation in computationally intensive kinetic models. Designed with a unique wrapper interface, petBOA supports both macrokinetic and microkinetic modeling tools, including Cantera and OpenMKM. By leveraging surrogate Gaussian processes through BoTorch, petBOA approximates and minimizes parameter estimation objectives, incorporating local and global sensitivity analyses to prioritize key parameters. Additionally, it utilizes pMuTT for consistent kinetic and thermodynamic parameters, allowing perturbations within conventional DFT error bounds.

## 3 Methods

A typical PINN consists of a neural network with an architecture that includes input layers, hidden layers, and output layers. The key innovation of PINNs is the incorporation of physical constraints into the network's loss function. Specifically, during training, PINNs optimize not only the error between the network's predictions and the observed data but also the residuals of the governing differential equations. This approach ensures that the network's predictions adhere to the physical laws described by the differential equations.

The training process involves minimizing a combined loss function, which typically includes a data loss term and a physics loss term. The data loss term measures the discrepancy between the predicted output and the observed data, while the physics loss term measures how well the network satisfies the differential equations that govern the system. By optimizing this combined loss function, PINNs can achieve solutions that respect both empirical data and physical laws.

PINNs can be employed in a variety of ways (Farea et al., 2024; Kim S. W. et al., 2021). Our primary focus here is on using PINNs to tackle both forward and inverse problems.

**Figure 1:**
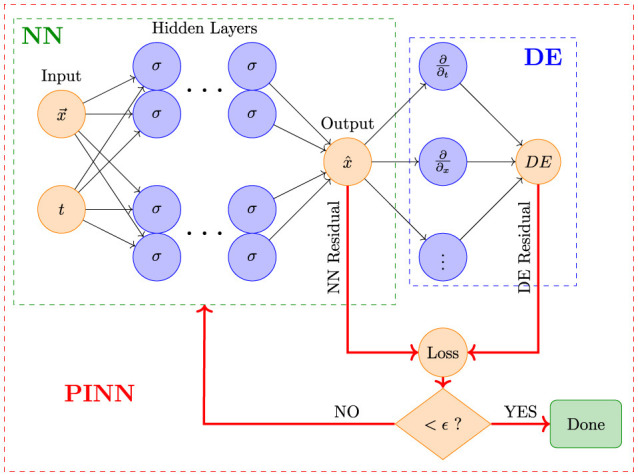
In the forward problem, PINN incorporates both the residuals from the Differential Equations (DE) and the data loss into the loss function to improve the accuracy of the Neural Network (NN). Here, ϵ represents the permissible margin of error.

The forward problem entails approximating solutions to ordinary or partial differential equations (DEs) by incorporating the governing physical laws into the loss function, as shown in Figure 1. It involves predicting the outcome or state of a system given known inputs and parameters. In the context of ODEs, a forward problem requires solving the differential equation to obtain the system's response over time. For instance, in a tumor growth model, the forward problem would involve using ODEs to predict how the tumor size evolves based on initial conditions and parameters. PINNs tackle forward problems by learning to approximate the solution to these differential equations, providing insights into the system's behavior under various conditions.

**Figure 2:**
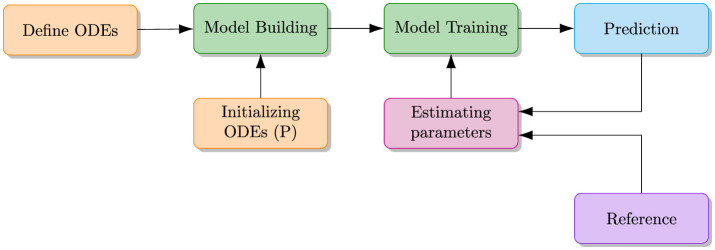
In the inverse problem, PINNs estimate the unknown parameters (P) of ODEs/PDEs by first providing an initial guess or starting values. They then minimize the difference between the predicted results and observed data throughout the training process to determine the true values.

Inverse problems, on the other hand, focus on estimating unknown parameters of ODEs/PDEs by minimizing the difference between predicted results and observed outcomes, as illustrated in Figure 2. In the context of ODEs, an inverse problem might involve determining the rate of tumor growth or the transmission rate of a disease from observed data. Solving inverse problems often requires fitting the parameters of the differential equations to match the observed data, which can be challenging due to the need for accurate parameter estimation.

PINNs are particularly well-suited for inverse problems because they can incorporate both the physical constraints of the differential equations and the observed data into their training process. By optimizing the parameters of the ODEs to minimize the discrepancy between the predicted and observed outcomes, PINNs can effectively estimate unknown parameters and improve our understanding of the system.

Overall, PINNs offer a powerful approach to solving both forward and inverse problems in dynamic systems by leveraging their ability to integrate physical laws into neural network training. This theoretical foundation enables PINNs to provide accurate and physically consistent solutions to complex problems modeled by ODEs, bridging the gap between data-driven methods and physical principles.

To gain a practical understanding of PINNs, the implementations of utilized ODEs-based models are outlined next sections. This hands-on approach will allow us to replicate these examples and build a foundation for applying similar techniques to related problems, which will be outlined. Before that we discuss some of the common details on the architecture, epochs and hyperparameter selection for the PINN implementations using DeepXDE.

### 3.1 PINN hyperparameters

The network architecture and training hyperparameters was selected based on network expressivity for PINN, numerical stability, and computational efficiency. However, it is not tested rigorously across different archtectirue for selecting best performing model. A fully connected internal layers are most common for PINN architecture. For the logistic growth model, we used 3 hidden layers with 50 neurons per layer; for the gene-expression model, four hidden layers with 50 neurons per layer. The *tanh* activation function was chosen to ensure smooth, differentiable outputs required for computing physics residuals. Learning rates are set to 0.001 for stable convergence with the Adam optimizer, and the number of training iterations (200,000 for logistic growth and 10,000 epochs for gene expression) was chosen based on initial testing to ensure that a stable convergence for forward and inverse problems both.

### 3.2 Loss function, convergence and stopping criteria

The total loss consists of three components: data loss (network predictions to observations), initial condition (IC) loss, and physics loss (ODE residuals at collocation points). All components are combined equally without explicit weighting. This simplifys training and avoid additional hyperparameters. The network automatically learns to satisfy the ODE, initial conditions, and data loss, while individual loss components may be larger than others at a given epoch or iteration, the network is trained for a sufficient number of epochs or iterations to ensure that all components steadily decrease and eventually converge toward zero. The initial monitioring shows that all losses decrease smoothly without a significant stagnation of any particular loss. In the case studies, we do not add any strict automated stopping criterion as the preliminary experiments showed that the chosen number of iterations (epochs) allows all the loss and inferred parameters to stabilize and a monotonic decrease indicates a convergence.

### 3.3 Collocation points

The time interval for example studies is *t*∈[0, 100]. To ensure the sampling rate is sufficient to capture all relevant dynamics of the ODE systems, the {500, 350} collocation points were used for the logistic growth and gene expression systems, respectively, drawn from a uniform distribution. These collocation points are sufficient because the example case studies of ODEs for population growth and transcription factor binding produce smooth, simple curves without discontinuities or oscillations. However, several studies provide adaptive and non-uniform sampling schemes for collocation sampling strategies (Sholokhov et al., 2023; Bauduin et al., 2025) which are useful for complex ODE systems. In such cases, a higher temporal resolution is required to capture the dominant dynamics of the system and for a finer spacing than the maximum time step allowed by the Nyquist-Shannon criterion (Ooi et al., 2024).

## 4 Software description

### 4.1 Case studies

To illustrate the practical implementation of PINNs, we discuss two case studies, rooted in biology and systems biology, both based on ordinary differential equations (ODEs) and systems of ODEs. The first example focuses on a population growth model, while the second examines a biological model of gene expression regulation. These examples offer hands-on experience to reproduce and extend solutions to similar problems, with extended versions of the code available on GitHub (Tripathi et al., 2024).

In the following, we focus on a particular type of differential equations which are given by a system of ordinary differential equations of the first order (Perko, 2013).

The general mathematical form is given by:


$$\begin{array}{l} \text{System of ODEs of }1\text{st order }\{\begin{array}{c} \frac{dx_{1}}{dt}=f_{1}\left(x_{1},x_{2},\ldots ,x_{n},t\right)\\ \frac{dx_{2}}{dt}=f_{2}\left(x_{1},x_{2},\ldots ,x_{n},t\right)\\ \vdots \\ \frac{dx_{n}}{dt}=f_{n}\left(x_{1},x_{2},\ldots ,x_{n},t\right) \end{array} \end{array}$$ (1)

where *x*_1_, *x*_2_, …, *x*_*n*_ are the state variables, and *f*_1_, *f*_2_, …, *f*_*n*_ are the functions defining the system dynamics. Such a system of ODEs is called first order because the derivative with respect to time is only of the first degree for each equation, i.e., $\frac{dx_{i}}{dt}$ for all *i*. There are numerous problems that can be described by systems of ODEs of 1st order because the functions *f*_*i*_ can be linear or nonlinear. Examples include the SIR model (epidemiology) (Martcheva, 2015), Hodgkin-Huxley Model (neuroscience) (McCormick et al., 2007), Lorenz system (meterology), Goodwin Oscillator (biology) (Ruoff et al., 1999), and Michaelis-Menten Kinetics (biochemistry).

### 4.2 Population growth model

The Logistic Growth Model is an example of a renowned differential equation that has been extensively utilized to describe population growth in various problems (Tsoularis and Wallace, 2002; Jin et al., 2018).

The Logistic Growth Model, also known as the logistic differential equation, is a first-order differential equation commonly used to model population growth in environments with limited resources. Its standard form is


$$\begin{array}{l} \frac{dP}{dt}=rP\left(1-\frac{P}{K}\right) \end{array}$$ (2)

where *r* represents the intrinsic growth rate, *K* is the carrying capacity and *P* is the current population size. One can see that for *n* = 1, *x* = *x*_1_ = *P* and $f\left(x,t\right)=f_{1}\left(x_{1},t\right)=rP\left(1-\frac{P}{K}\right)$ one obtains Equation 1. The analytical solution of Equation 2 is given by


$$\begin{array}{l} P\left(t\right)=\frac{K}{1+\left(\frac{K}{P_{0}}-1\right)e^{-rt}}, \end{array}$$ (3)

where *P*_0_ is the initial population at *t* = 0.

The Logistic Growth Model consists of two components. The first component, given by *rP*, represents the exponential growth of a population, controlled by *r* is the intrinsic growth rate. The second component, −*rP*^2^/*K*, represents the reduction in growth as the population approaches the carrying capacity *K*. This term reflects a negative feedback mechanism where population growth decreases proportionally to the population size relative to *K*. The resulting S-shaped form of P is similar to a logistic function explaining the name of the model. In general, the logistic differential equation effectively models the dynamics between population growth and environmental limits, making it a valuable model in ecology (Gamito, 1998), epidemiology, agriculture (Holzinger et al., 2024), and economics (Smirnov and Wang, 2020).

#### 4.2.1 Inverse problem

In this section, we address the inverse problem, which involves estimating unknown parameters of ordinary differential equations (ODEs) by minimizing the physics loss.

**Figure 3:**
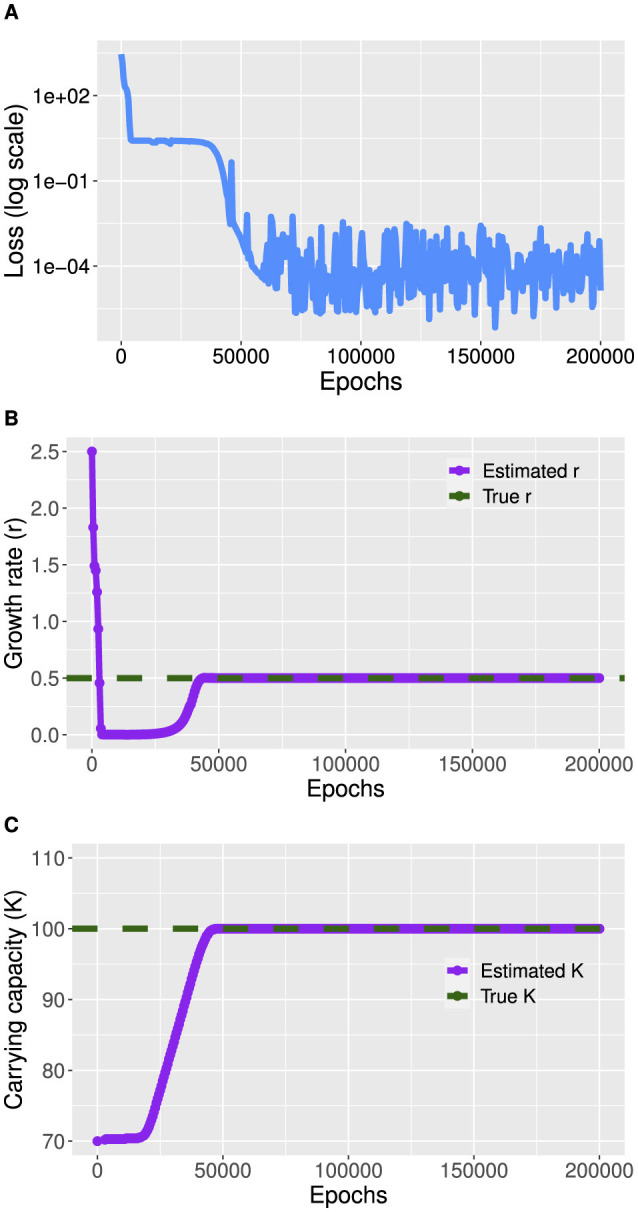
Training of the logistic growth model using the code in Listing 1. The true values of the parameters are *r* = 0.5 and *K* = 100. **(A)** Average loss function. **(B)** Optimization of r. **(C)** Optimization of K.

In Listing 1, we provide an implementation utilizing DeepXDE, a widely used Python package (Lu et al., 2021). For a better understanding, we substructure this problem into 10 steps discussed below, each corresponding to a part of Listing 1. Executing these parts consecutively will give the final output. A visualization of the loss function and the parameter estimation is shown in Figure 3. For a better reproduction, the code can also be found on GitHub (Tripathi et al., 2024).

Listing 1 **Population growth model: inverse problem.**

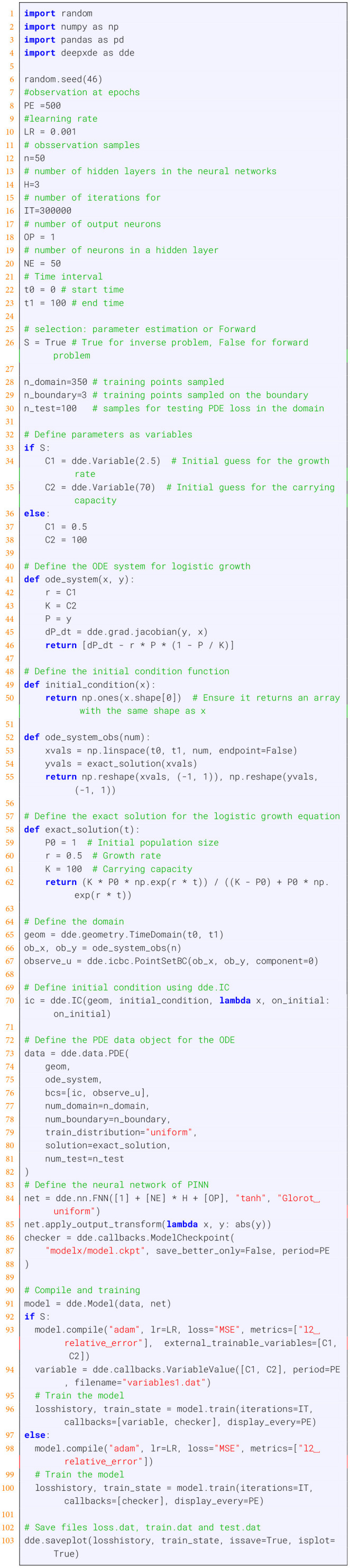

#### 4.2.2 Implementation steps

Implementing the Logistic Growth Model as an inverse problem using PINNs involves several key steps. These steps can be outlined as follows:

1. Importing packages and parameter settings: In line 1 to 4 the required packages are loaded and in lines 7 to 30 we set all parameters. Importantly, the logic variable S (selection) allows to set the inverse problem (True) or forward problem (False) discussed in Section 4.2.3.
2. Defining parameters as variables: For the inverse problem, one needs to define which parameters of the ODE should be learned during the training. For the population growth model, we have two parameters and in line 34 and 35, we set these as trainable variables.
3. ODE system and initial conditions: The ODE system of the population growth model is defined in lines 41 to 46 and the corresponding initial conditions are defined in line 49 to 50. The initial conditions are set in lines 70 and 71.
4. Observational data: For the training, the model needs observational data. In real-world examples these may come from measurements. However, in our case, we generate these observational data from the true model. For this reason, we define how the data are sampled (lines 52 to 55) and the exact solution of the population growth model (lines 58 to 62).
5. Define the domain: To specify the range of values for the independent variables over which the differential equation is solved. For ode, the spatial and temporal domains can be specified using methods dde.geometry.Interval, dde.geometry.TimeDomain. Additionally, initial and boundary conditions are specified to ensure that the solution to the ODE meets the necessary constraints. Initial conditions define the solution at the initial point of the independent variable, and boundary conditions define the solution at the boundaries of the domain or at specific points of the independent variable.
6. Define PDE data object: is created to define and configure the specified problem for solving. The deepxde.data module is applied to define the data and problem in deepxde. The inputs are domain information, ode function, initial and boundary conditions, and sampling data in the domain and at the boundaries.
7. Define neural network of the PINN model: First, the architecture and the type of the NN are defined (line 84). In our example, the NN has one input (*t*), H hidden layers and NE neurons on each. Finally, the last layer has OP output neurons. The output_transformation (line 85) ensures rescaling and transformation of the output and avoiding any normalization or scaling by neural network output and complying to the constrained of the ode systems The callback function dde.callbacks.ModelCheckpoint saves the state of a trained model at specified intervals during the training process, which is useful for long training sessions, as it helps to save progress while training the model. It can also help recover the model from previous states if training is interrupted. Other than that, it evaluates the model's performance at different stages by saving various versions of the model throughout the training process.
8. Compile and training of the PINN shown in lines 93 to 96: For the inverse problem, the steps involve setting up trainable parameters that are to be optimized during training and compiling the model with these parameters. These parameters include a list of variables initialized as dde.Variable objects based on initial guesses. Additionally, preparing the model for compilation requires specifying various input parameters and hyperparameters, such as the optimizer, learning rate, loss function, and metrics. For the training steps, it is necessary to provide the number of iterations (epochs) and a list of callback instances. Callbacks include functions like ModelCheckpoint for saving model states and VariableValue for monitoring the states of parameters that are being optimized, and other custom callbacks for monitoring the training process.
9. Generate Predictions: After training, predictions are by using the test data shown in Listing 2. The data can be user defined or the testing data used for testing in the training process from the data object in line 73 of the “data” object in Listing 1. It is important to note that to load a checkpoint, the model object from training must still exist in memory; if not, the model object needs to be reinitialized with the same architecture and compile settings.
10. Plotting of the results: Finally, the results are visualized in Figure 3 as produced by Listing 3.

**Figure 4:**
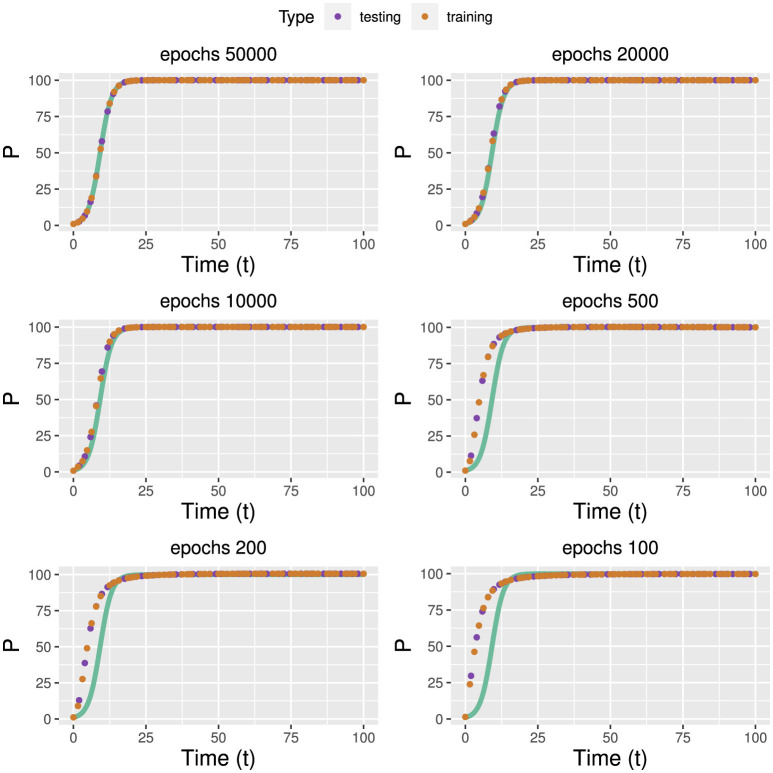
Training of the population growth model for different epochs for the forward problem. The green curves corresponds to the true model.

Listing 2 **Predictions the output “P” over epochs, see Figure 4.**

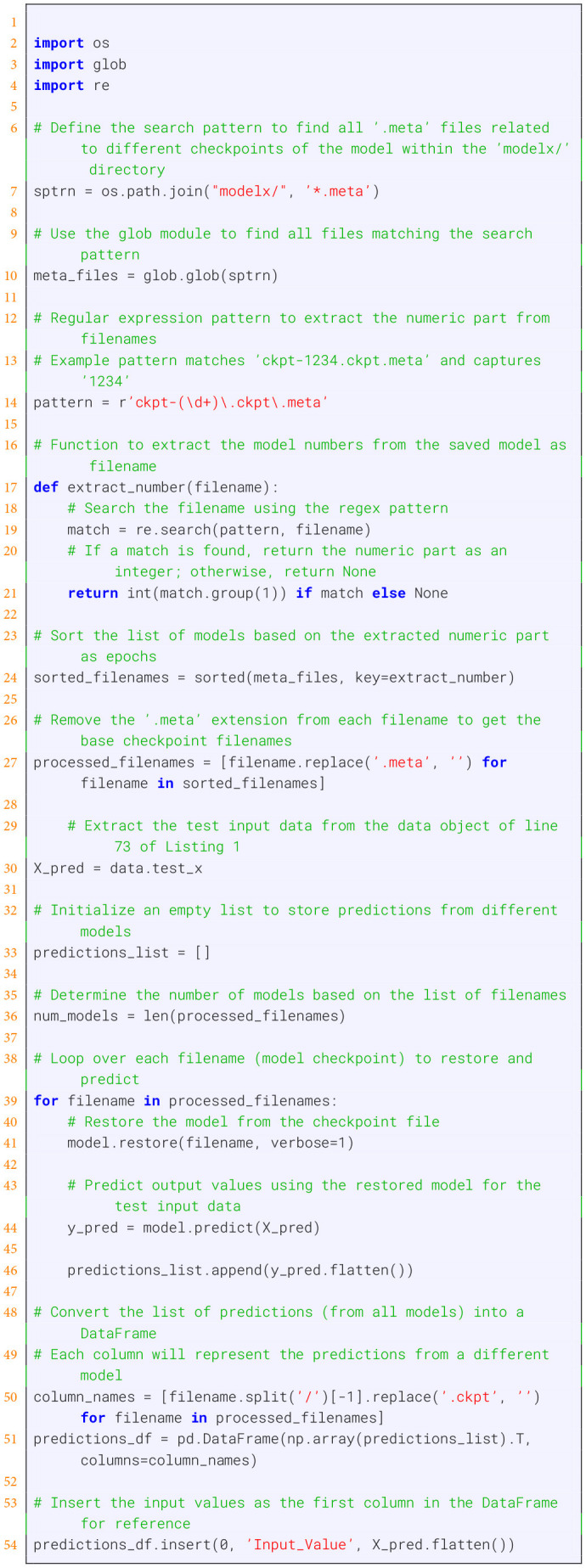

Listing 3 **Visualizing the parameter optimizations over epochs, see Figure 3.**

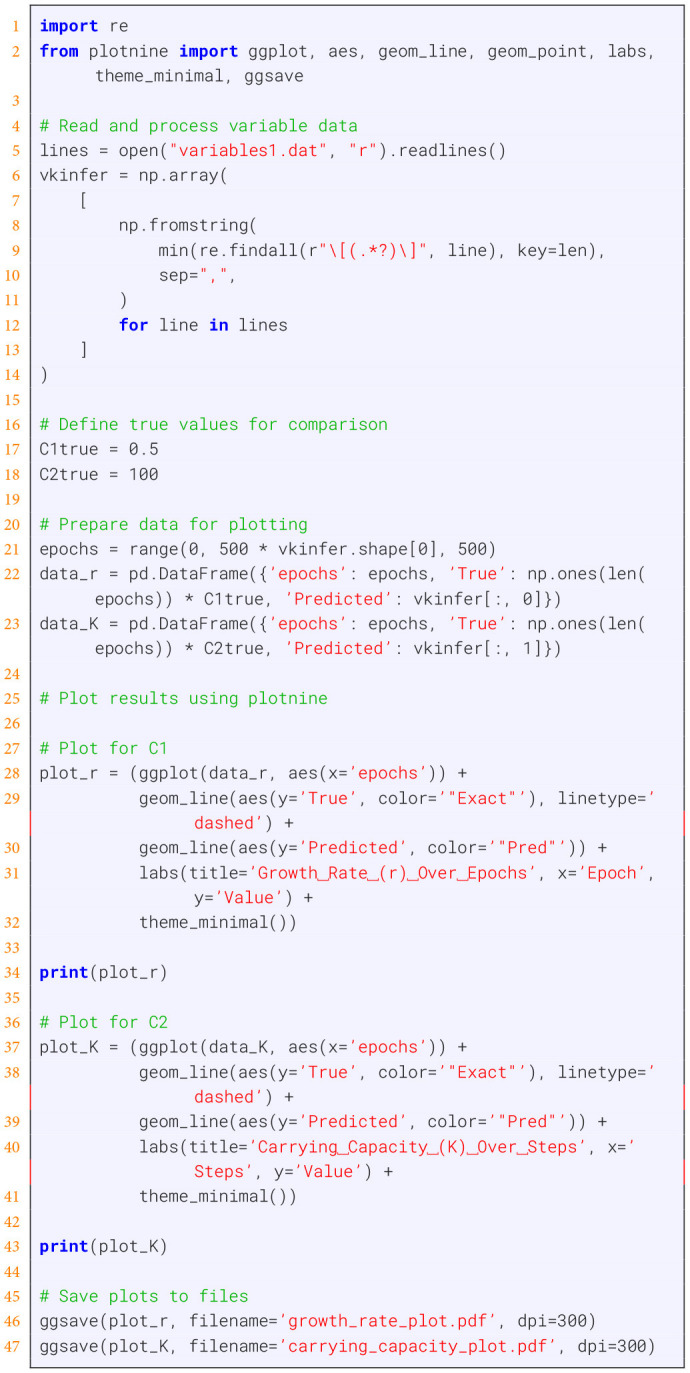

#### 4.2.3 Forward problem

To see the similarity between the forward problem and the inverse problem, we wrote Listing 1 in a way that allows an easy transition. Specifically, only lines 8 to 31 in Listing 1 need to be replaced by Listing 4. These are just different parameter configurations appropriate for the forward problem. All other changes are invoked by setting S = False.

Listing 4 **Parameters for the forward problem for the population growth model.**

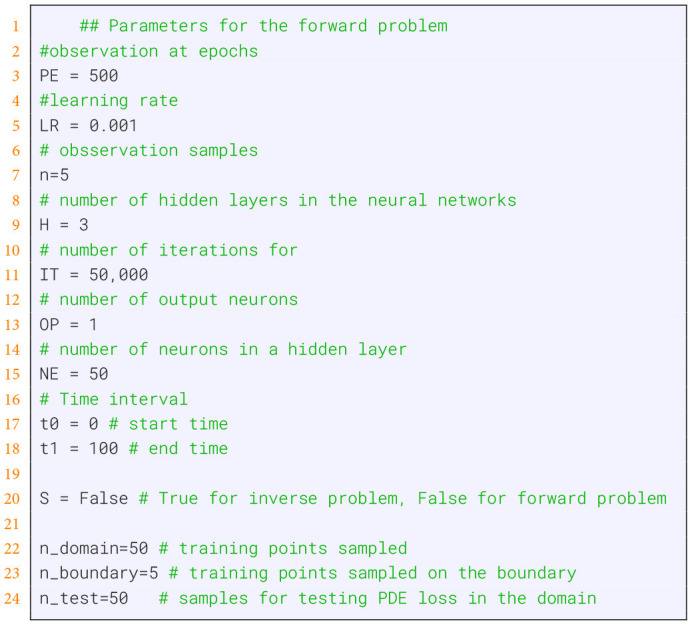

The results of the training of the forward solution for different epochs are shown in Figure 4. In these figures, the green curves corresponds to the true model whereas the red and purple points correspond to training respectively testing data. Overall, one can see that the model converges quickly and already 10,000 epochs give good results.

#### 4.2.4 A comparsion with the neural network

In this section we provide a comparsion of the PINN with the standard neural network approach with the PINN of the population growth model. In order to make a fair comparison where the usefulness of PINN can be highlighted we implemented both the model with torch library in python rather than using DeepXDE. For the comparsion, the true parameters are set to *r*_*true*_ = 0.5 and *K*_*true*_ = 100.0. The initial population is *P*_0_ = 1.0. First, we generate n={10, 15, 20, 30} synthetic, noisy observed data points (*y*_*obs*_) over a time span of *t* = 0 to *t* = 100 using the known analytical solution to the ODE. The first section trains a Standard Neural Network (NN Model). This model is a simple feedforward network with three hidden layers, trained using the adam optimizer and a mean squared error (*MSE*) loss function. The loss is calculated only between the predictions and the observed noisy data (*y*_*obs*_). This approach treats the problem as a data-fitting modeling and does not incorporate the physics loss. The PINN model has a similar structure to the standard NN, but it contains two additional, key parts: the physical parameters *r* and *K* are implemented as trainable parameters, and the loss function is composed of multiple parts includes the data loss, the initial condition (IC) loss (enforcing *P*(0) = 1), and the physics loss. The physics loss is calculated at 200 collocation points (*t*_*colloc*_) spanning the time domain. We used automatic differentiation (torch.autograd.grad) to compute the time derivative of the population (*dP*/*dt*) predicted by the neural network. The loss then minimizes the squared residual of the ODE, making the prediction to satisfy the ODE Equation 2. The implementation for comparison is shown in the Listing 5. For the evaluation of the outcome the predictions of both the standard NN and the PINN against the true analytical solution over an extended time range (*t* = 0 to *t* = 150, demonstrating extrapolation) calculating mean squared error (*MSE*) of each model's prediction against the true system and estimated *r* and *K* by the PINN for different samples of observed data. The results of the comparison is shown in the Table 1 where PINN performs consistently better than the standard neural network when the number of samples are small (*n* = 10 to 20), with lower data points, the NN has large errors, while the PINN shows less error because it uses the physics loss as a constraint. The PINN also closely estimates the true model parameters even with limited data. As the sample size increases (n = 30 to 50), both models improve, while the NN can fit the data reasonably well, the PINN consistently recovers the correct parameters capturing the underlying dynamics of the system.

Listing 5 **Comparison between NN and PINN.**

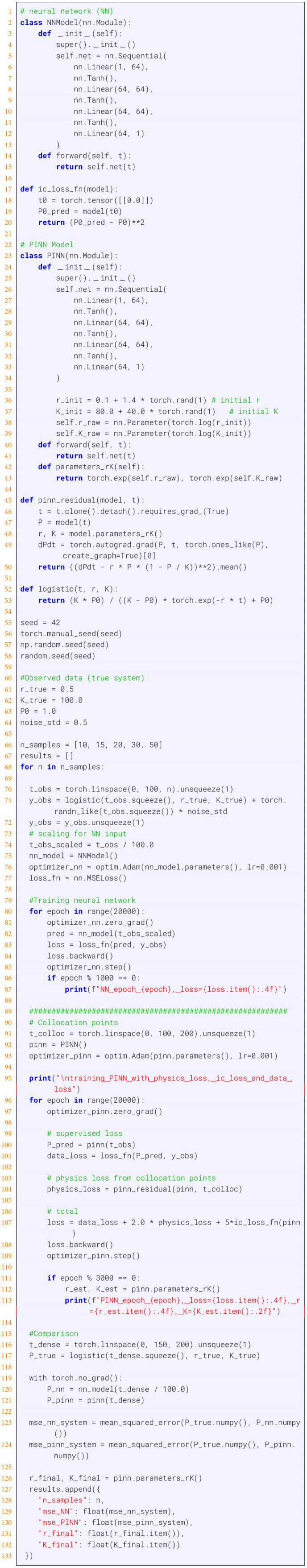

**Table 1:** NN vs. PINN performance and parameter estimates at different sample sizes.

***Samples*(*n*)**	**MSE NN**	**MSE PINN**	** $\hat{r}\left(PINN\right)$ **	** $\hat{K}\left(PINN\right)$ **
10	16.06	3.38	0.39	100.09
15	1.84	0.12	0.49	99.86
20	0.99	0.016	0.50	99.97
30	0.064	0.024	0.49	100.04
50	0.035	0.044	0.49	100.01

### 4.3 System of ODEs for gene expression

The next case study is on the regulation of gene expression by transcription factors given by a system of ODEs (Polynikis et al., 2009; Bolouri and Davidson, 2002).

Such a biological system involves interactions between three components: a promoter (*prom*), a transcription factor (*tf*), and their complex (*promtf*). These interactions depend on the rate constants *k*_on_ (the binding rate) and *k*_off_ (the unbinding rate). In the following, we focus on estimating the parameters of the system.

The system of ODEs for gene expression regulation is described by the following equations:


$$\begin{array}{l} \frac{d\left(\text{prom}\right)}{dt}=-k_{\text{on}}\cdot \text{prom}\cdot \text{tf}+k_{\text{off}}\cdot \text{promtf} \end{array}$$ (4)

$$\begin{array}{l} \frac{d\left(\text{tf}\right)}{dt}=-k_{\text{on}}\cdot \text{prom}\cdot \text{tf}+k_{\text{off}}\cdot \text{promtf} \end{array}$$ (5)

$$\begin{array}{l} \frac{d\left(\text{promtf}\right)}{dt}=k_{\text{on}}\cdot \text{prom}\cdot \text{tf}-k_{\text{off}}\cdot \text{promtf} \end{array}$$ (6)

Here the dynamics are for the rate of change of the promoter (*prom*) (Equation 4), transcription factor (*tf*) (Equation 5) and promoter-transcription factor complex (*promtf*) (Equation 6).

For our case study, we set the true parameters for *k*_on_ and *k*_off_ to 1/60 and 0.5/60, respectively. The initial conditions for the system for *t* = 0 are given by [17.0, 25, 0], representing the initial concentrations of prom(0), tf(0), and promtf(0). To generate synthetic data, the ODE system is solved numerically over a time range from 0 to 100 with 300 time points. For this, the true parameters and initial conditions given above are used.

The implementation of the Physics-Informed Neural Network (PINN) for estimating the parameters is shown in Listing 6. Initial estimations for *k*_on_ and *k*_off_ are set randomly.

Listing 6 **Gene expression regulation by transcription factors.**

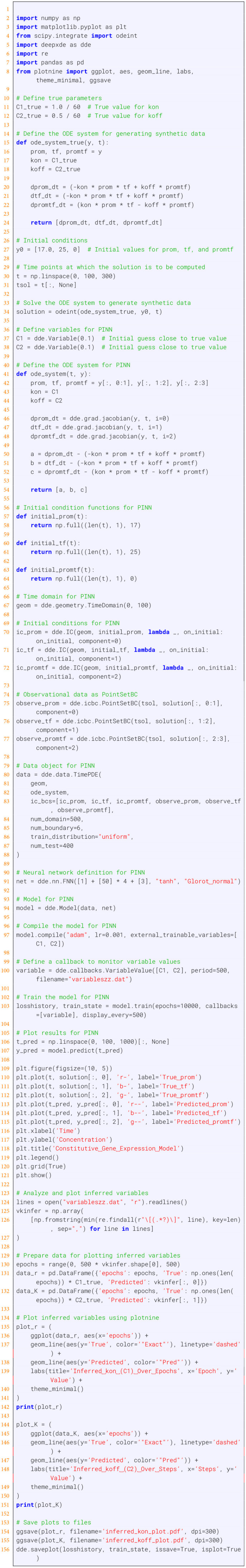

**Figure 5:**
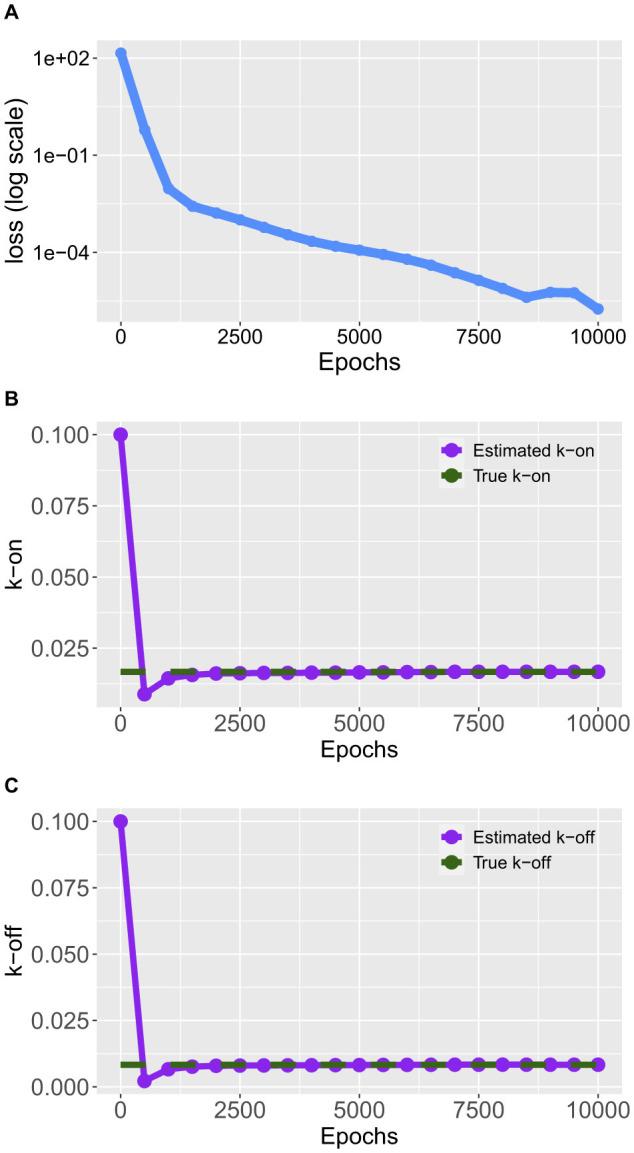
Training of the gene expression model in Listing 6. The true values are *k*_*on*_ = 0.0166, and *k*_*off*_ = 0.0083. **(A)** Average loss function. **(B)** Optimization of the parameter *k*_*on*_. **(C)** Optimization of the parameter *k*_*off*_.

For our example, we define a feedforward neural network, taking time *t* as input and the resulting output is given by the concentrations of *prom, tf*, and *promtf*. The PINN integrates the physics loss function, which computes the gradients of the output variables with respect to time and ensures these gradients match the right-hand sides of the ODEs. Figure 5A shows the training loss over time.

During the training phase, the PINN model adjusts its internal parameters, including *k*_on_ and *k*_off_ shown in Figures 5B, C, to minimize the difference between its predictions and the synthetic data. A callback function monitors the values of *k*_on_ and *k*_off_ throughout the training process to track their evolution and convergence.

By using a Physics-Informed Neural Network (PINN), we can accurately estimate the parameters *k*_on_ and *k*_off_ of a biological system modeled by ODEs. The PINN leverages both the observational data and the underlying physics (ODEs) to improve the predictions, providing a powerful tool for parameter estimation in ODE systems.

We would like to emphasize that the initial values of the parameters of the model, including the weights of the PINN and the parameters of the system of ODEs, affect the convergence process during training. Accurate initial guesses can help the PINN to quickly find the optimal solution by improving optimization efficiency. Conversely, if the initial guesses are far from the true values, the training process may require more iterations to converge, leading to slower learning. Therefore, reasonable estimates for the initial parameters can facilitate faster and more efficient convergence in the optimization process.

#### 4.3.1 Forward problem

**Figure 6:**
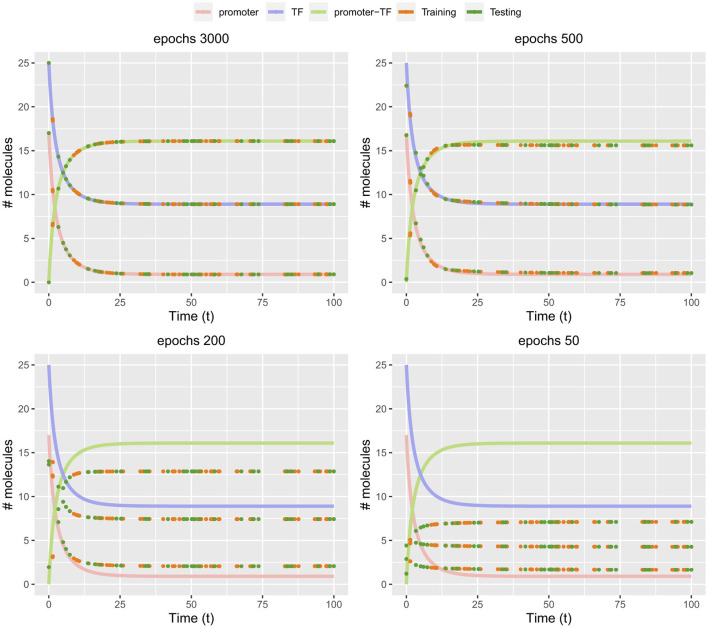
Actual, training and predicted values of promoter, transcription factor and promoter-transcription factor of Listing 6 at different epochs.

Similar to the population growth model, also for the gene expression model, we obtain the solution to the forward problem from a modification of the code. The corresponding listing can be found on gitbub. In Figure 6, we show the numerical results for the gene expression model. As one can see, the at the beginning of the training, up to 200 epochs, the predictions of the PINN are poor. However, when further increasing the number of epochs, convergence is reached after about 3000 epochs.

On a general note, we would like to emphasize that in our analysis for this and the preceding case study, efficiency was not our primary concern. Instead, we focused on the fundamental structure of PINNs and their basic understanding. However, for practical applications, training convergence is crucial, as complex systems can otherwise demand a significant amount of training time.

### 4.4 Discussion

After presenting our case studies, we want to discuss general issues and challenges when training PINNs for ODEs or systems of ODEs by using DeepXDE.

1. Definition of the loss functions: DeepXDE does not provide an option for explicitly defining individual loss functions that are part of the total loss. Instead, they are automatically invoked once certain components are added. For instance, definition of an ODE system (def ode_system) leads to the inclusion of a physics loss while the definition of an initial condition (def initial_condition) leads to the inclusion of a loss for initial condition. While these terms cannot be added individually, one can specify the functional form of a loss function (metrics).
2. Access to the physics loss function: To solve the inverse problem for estimating the parameters of the dynamical system, DeepXDE optimizes the physics loss during the learning of the PINN. However, the physics loss as a function of the parameters cannot be directly accessed during this learning process, nor is it possible to specify alternative optimization methods for it. Direct access to the physics loss would mainly facilitate adaptive training strategies where detecting stagnation in the ODE residual may allow to adjust collocation points, loss weights, or optimizers. Implementing such adaptive training would require customized callbacks or functions to modify the training procedure.
3. Initial and boundary conditions: For specifying a differential equation, initial conditions (IC) or boundary conditions (BC) are required. The initial conditions and boundary conditions are provided under the “icbc” class providing different classes and functions to define and handle the initial and boundary conditions for differential equations. Specifically, the initial conditions are set by classes and functions within the module deepxde.icbc.initial_conditions, and the boundary conditions are defined similarly using the classes and functions in the deepxde.icbc.boundary_conditions module. This ensures that both initial and boundary conditions are properly specified for solving differential equation problems. Additionally, for the inverse problems or for observed data, user can use dde.icbc.PointSetBC function to incorporate these observations. Function deepxde.icbc.OperatorBC allows to incorporate custom boundary conditions involving derivatives or other operations on the solution. Importantly, such definitions add automatically a corresponding loss term to the total loss be considered by the training of the PINN.
4. Weighting of individual losses: The total loss for training the PINN is a weighted sum of the individual losses. The weights can be set manually by using the loss_weights option in the process of configuring and preparing the model for training. If this is not specified equal weights are assumed. Additionally user can provide different loss functions under loss option for different losses.
5. Observational data: The model uses observational data for training the PINN. In our example for the logistic growth model, we assumed to know the analytical solution (exact_solution) for sampling observations (ode_system_obs) to obtain such data (data). This is a special case because in many situations the exact solution may not be available. For such cases, we could either receive directly such observations, e.g., via measurements from an experiment, or generate them indirectly via numerical simulations instead of the analytical solution. Both alternatives require the adjustment of the code in Listing 1.
6. Definition of the network architecture: A PINN requires a particular architecture of the neural network that needs to be user-defined. For this, DeepXDE provides several options including Fully Connected Neural Network (FNN), Residual Neural Network (ResNet), Multiscale Fourier Feature Networks, Stacked FNN for a customizable way for specific requirements for the differential equations to be solved. The performance and accuracy of the PINN can significantly depend on the neural network architecture.
7. In DeepXDE, data transformation is a useful step for solving differential equations with PINNs. The transformations help in scaling inputs, and adapting the training process. User can utilize custom functions for transformations to preprocess inputs to improve the PINN's performance for differential equations. The user can define customizatble transformation functions that preprocess inputs to improve the neural netwtwor's performance. The member functions apply_output_transform and apply_feature_transform of defined neural network instance in DeepXde can be utilized for input data transformation for neural network in DeepXde.
8. The compile and train functions of the deepxde.Model class offer several flexibe options for model training. The compile method allows for the configuration of various training parameters including optimizer selection and configuration, learning rate, loss functions, loss weights assignment for different losses, and an option to include additional external trainable variables (external_trainable_variables) that are not part of the neural network's architecture. This is needed for inverse problems where parameters of the differential equations are required to be estimated from the data.
  The train method allows the execution of the training process with customizable parameters such as the number of iterations, batch sizes, and the frequency of progress updates. It also allows for control over the training process with options to accept or reject previous best models, apply callbacks for additional control and monitoring, and specify paths for model restoration and checkpoint saving. These features help in managing and optimizing PINN training in DeepXDE.
9. The Callback class in the deepxde.callbacks module provides several functions to monitor and control the training process of PINNs. Callbacks allow to execute specific functions at different stages of the training process.
10. The loss history and train state objects of the LossHistory and TrainState class in DeepXDE are returned in the training process which are essential for monitoring and analyzing the training process of PINNs. The losshistory provides a record of the loss values over epochs, while train state provides detailed information about the model's state during and after training. These tools help in diagnosing issues, and optimizing the training process.
11. In case the initial or boundary conditions are not known, the user can specify them as an empty list input ([]) in the steps of the PDE data object creation. This assignment allows the model to be defined and solved without these conditions. In this case, the PDE data object will be set up without initial or boundary conditions for solving the differential equation itself.

From this discussion it becomes apparent that Physics-Informed Neural Networks (PINNs) are more complex to train, understand, and interpret compared to traditional deep learning neural networks. A reason therefore is that training PINNs requires expertise in both differential equations and optimization, and a technical understanding of neural network models. This involves also incorporating multiple types of loss functions to ensure that the network adheres to the constraints imposed by the differential equation model. In the example studies we used the Adam optimizer for its robustness in training PINNs with and its ability to achieve faster initial convergence for our relatively simple one-dimensional cases. While other optimizers available in DeepXDE (LBFGS, or other advanced optimizers only for specific backends) with hybrid strategies improve convergence speed or final accuracy. Rathore et al. (2024) shows PINN training is affected by ill-conditioned loss landscape, which can be mitigated by ADAM and L-BFGS. In this approach Adam initially roughly minimize the loss and then a switch to L-BFGS, which further improves convergence of all loss components. This is effictive even in cases involving ill-conditioned differential operator, where small changes on neural network weights leads to a large changes in the residual loss.

As seen from our presentation of the case studies, the DeepXDE package is complex containing a lot of details to specify a PINN solution. Unfortunately, the documentation for various options is sometimes lacking sufficient details. Also, more flexibility would be desirable with various options for users to specify different variants. In this context, it should be noted that several limitations of DeepXde have already been discussed, e.g., in Lu et al. (2021). For example, DeepXDE does not support complex curvilinear boundaries.

Other issues are setting up initial weights (DeepXde, 2024b). Also the batch_size option is not fully implemented which can make training of large data slow and inefficient (DeepXde, 2024a). Several other problems often faced by the users relate to training stability and convergence issues. Lastly, there can be also compatibility problems with the latest updates of various dependent Python packages. While this is not strictly related to DeepXDE, it can cause practical problems when setting up an implementation.

Overall, DeepXDE serves as a solid starting point for using default PINN configurations. However, it lacks some flexibility for experimenting with novel ideas and deviating from the standard framework. Therefore, DeepXDE is best suited for studies that aim to apply standard PINNs in various application domains. As demonstrated in our case studies, ODEs and systems of ODEs can be implemented, which should have numerous applications beyond the physics domain.

Finally, we note that parameter identifiability is an important general aspect of inverse problems for dynamical systems, as it determines whether model parameters can be uniquely inferred from available observations (Wieland et al., 2021). In many practical situations, different parameter values may lead to similar system behavior, resulting in non-identifiable or weakly identifiable models. This issue is particularly relevant when data are limited, noisy, or only partially observed. In the absence of explicit identifiability analysis, estimated parameters should therefore be interpreted with caution, since multiple solutions may be consistent with the data. Addressing identifiability through analytical methods, sensitivity analysis, or numerical experiments is essential for assessing the reliability of parameter estimates.

## 5 Future directions

In this section, we will discuss a few desirable extensions of DeepXDE that could add more flexibility to analyses. Currently, these extensions are not available in DeepXDE.

An important extension of PINNs would be Neural ODEs, which can be viewed as their close relative. Neural ODEs represent the continuous limit of residual or recurrent networks, as described by Chen et al. (2018). They model continuous transformations of the latent state by parameterizing the derivative of the unknown function, unlike PINNs. This derivative is then passed to a numerical ODE solver for integration, with the solver's output serving as input to the loss function. Backpropagation through the solver is achieved via the adjoint sensitivity method, as introduced by Pontryagin (1987).

Lastly, we would like to highlight that PyTorch's ecosystem addresses many of the challenges mentioned earlier. Specifically, torch.func (PyTorch, 2024) enables the functionalization and differentiation of the forward pass of a PyTorch module with respect to both inputs and parameters. This provides a flexible framework for implementing PINNs in Python while retaining the versatility of PyTorch in defining models and loss functions. Additionally, libraries like torchdiffeq (Chen, 2018) and torchdyn (Poli et al., 2021) offer robust implementations for Neural ODEs.

Additionally, the ODE challenges for biological systems need to be accounted for in PINN-based frameworks such as PyTorch libraries or DeepXDE. Several of these challenges are related to stiff ODEs (Kim S. et al., 2021; Kainth et al., 2025), for which output scaling, stabilized gradients, and appropriate loss functions are required to address computational instabilities in effective forward and inverse ODE problems. Similarly, spectral bias is another challenge when the neural network learns low-frequency features more smoothly than the high-frequency features, requiring approaches such as spectral-aware training (Ahmadi et al., 2025). Parameter uncertainty and non-identifiability are other challenges for PINN-based estimation. The non-identifiability issues (Giampiccolo et al., 2024) show that PINN-based solutions for biological systems face several challenges that go beyond standard deep learning problems and require careful evaluation of the network design and training strategy, along with ODE system insights (Lagergren et al., 2020). In line with this, it is important to systematically consider the existing literature covering the application of PINNs across different domains, discussing various challenges associated with their implementation. While these challenges are often discussed within the context of a particular domain, they need to be comprehensively understood in a general context. This understanding will enable the development of a general applicability for PINN based implementation package. By consolidating several solutions to the challenges discussed in the literature into modules, functions, or methods, one could create PINN packages that are practical and scalable for use across diverse applications.

## 6 Conclusion

Physics-informed neural networks (PINNs) represent a significant advance in the integration of deep learning with physical laws and has an advantage over neural networks. By embedding governing physical equations directly into the architecture of neural networks, PINNs enhance the model's capacity to tackle complex problems, particularly in situations with limited data, where traditional neural network models would typically need extensive training datasets. This paper focuses on the inverse problem, as many challenges involve parameter estimation of ordinary differential equations, which is achieved by minimizing the physics loss.

The implementation of PINNs is complex and requires technical proficiency in programming. Therefore, we provided a detailed description of a Python implementation using DeepXDE. We included case studies for ordinary differential equations and systems of ODEs, as many applications outside the physics domain rely on such models. The code provided in our examples should facilitate easy adaptation to similar problems involving ODE systems. This could serve as a solid starting point for other studies, particularly in biology, epidemiology, economics, life sciences and engineering.

## Data Availability

The original contributions presented in the study are included in the article/supplementary material, further inquiries can be directed to the corresponding author.
